# The Uncommons: A Case of Pancreatitis and Hepatitis Complicating Salmonella Infection

**DOI:** 10.7759/cureus.26422

**Published:** 2022-06-29

**Authors:** Tahani Almohayya, Hattan Alhabshan, Lana Alhouri, Hussam Al Hennawi, Ali Alshehri

**Affiliations:** 1 Department of Pediatrics, Dr. Sulaiman Al Habib Medical Center, Riyadh, SAU; 2 Department of Pediatrics, Alfaisal University College of Medicine, Riyadh, SAU; 3 Department of Internal Medicine, Alfaisal University College of Medicine, Riyadh, SAU

**Keywords:** lipase, elevated amylase, acute pancreatitis (ap), non-icteric hepatitis, salmonella gastroenteritis

## Abstract

*Salmonella typhi* infection can be associated with serious complications, ranging from self-limited to fulminant organ damage. In particular, liver and pancreatic damage may complicate the course of infection resulting in devastating outcomes. Enteric fever encompasses a tropical disease caused by *Salmonella*species and can be associated with high morbidity and mortality. Invasive infection rarely presents with acute hepatitis and pancreatitis. Early recognition of associated clinical conundrums can improve prognosis in affected patients. Here, we present a case of acute hepatitis and pancreatitis in an otherwise healthy child.

## Introduction

Typhoid fever, interchangeably termed enteric fever, underlines a systemic infection caused by *Salmonella enterica* serovar *typhi* (*S. typhi*) or by the less virulent subtype *S. paratyphi*. The infection remains a challenge in developing countries, with sporadic cases of typhoid fever still identified globally [1]. Rarely, multiple organs, including the liver and the pancreas, may concurrently be involved in the course of infection, resulting in serious complications which may hinder the prognostic outcomes of affected patients [2]. Indistinguishable from other disease entities, non-typhoid fever may present similar to acute gastroenteritis and acute viral hepatitis [3].

Non-typhoidal salmonellosis is characterized by a self-limiting acute episode of acute enterocolitis in most patients [4]. Clinical presentation may differ according to the patient’s overall health and may present as an invasive non-diarrheal febrile illness, bacteremia, meningitis, and focal infection with significant morbidity and mortality [5,6]. Of note, patients at risk include infants and children, the elderly, and immunocompromised patients whose immune systems may fail to react to an invasive infection [7]. The global burden of diseases study in 2017 estimated a total of 535,000 cases of invasive non-typhoidal *Salmonella* infection, of which 77,500 were fatal [8]. Most non-typhoidal invasive disease illnesses tend to occur in Sub-Saharan Africa where non-typhoidal salmonellosis represents a leading cause of community-acquired bloodstream infection [9,10]. According to Marchello et al., the overall reported incidence of invasive non-typhoidal infection is 100 cases per 100,000 individuals per year in some regions of Sub-Saharan Africa and 1,000 cases per 100,000 in children in the geography [11].

Clinically, invasive non-typhoidal infections may present similar to different febrile illnesses, rendering early recognition and timely treatment cumbersome [12]. The sequelae of invasive non-typhoidal infection may include distant bacteremia resulting in sepsis, osteomyelitis, septic arthritis, and pulmonary infections [6,7]. To date, greater than 2,500 serovars of *S. enteritica *have been identified with two serovars, Typhimurium and Enteritidis, constituting the most invasive non-typhoidal infections in humans [13,14]. To determine definitive isolates and antimicrobial susceptibility, one should consider blood, cerebrospinal fluid, and bone marrow, and sterile sites culture is suggested, which may pose a challenge in low-resources countries [6]. Of note, isolates with multidrug resistance to third-generation cephalosporins have emerged, hindering appropriate management of invasive non-typhoidal infections and the possible deterioration of severe illnesses when administered [15]. Owing to the challenge in controlling severe infections, considering an efficacious vaccine that is the target of the vacc-iNTS research project would add tremendously to the efforts to limit invasive non-typhoidal infections in the future [16,17].

Both hematogenous and lymphatic spread are currently the proposed mechanisms underlying pancreatitis, with direct penetration and migration of *Salmonella* from the gastrointestinal tract to the pancreatic duct remaining another possible route. Host immune reaction and toxin-mediated effects have also been theorized to explain possible *Salmonella* pancreatitis and hepatitis [18,19]. Higher relapse rates were observed in patients with hepatic involvement complicating *Salmonella* infection [4]. Although hepatomegaly and mildly elevated liver enzymes can parallel the disease course, acute hepatitis remains a rare complication. Rarely, pancreatic involvement may be added to the clinical picture and complicate an underlying *Salmonella* infection with various severities. Acute pancreatitis has been reported in adult cases with either typhoid fever or non-typhoid *Salmonella* infection. Nevertheless, few cases have been reported in the pediatric population [5]. We present a case of acute hepatitis and pancreatitis in an otherwise healthy patient.

## Case presentation

A five-year-old girl with no significant medical history presented with a two-day history of high-grade fever, associated with frequent vomiting, diarrhea, and abdominal pain. Vomiting was not projectile, nor was it bilious or mixed with blood or mucous. The diarrheal stool was free of any mucus or blood. The abdominal pain was mild and colicky, limited to the periumbilical area with no radiation. The pain was relieved by passing stool but aggravated by food intake. The patient did not complain of abdominal distension, chills, or rigors. She denied any history of drug ingestion or recent travel. Upon further questioning, the parents revealed a history of eating shrimp from an outside restaurant. A thorough physical examination on admission revealed a febrile child with a temperature of 39°C, along with a heart rate of 144 beats per minute. The patient showed signs of moderate dehydration. The rest of the physical examination, including a thorough abdominal examination, was unremarkable.

Laboratory investigations revealed a normal complete blood count with normal renal function. Urine analysis revealed elevated ketones with negative nitrate and leukocytes (Table 1). The patient had a decreased bicarbonate level with an otherwise normal electrolyte panel. Blood, stool, and urine cultures were obtained. Based on the presenting symptoms, the patient was admitted as a case of acute gastroenteritis presumptive of food poisoning. She was started on intravenous (IV) hydration, ondansetron, oral metronidazole, and paracetamol. Due to continued fever, antipyretic was switched to ibuprofen.

**Table 1:** Laboratory findings during the patient’s course of hospitalization. WBC: white blood count; RBC: red blood cell; Hgb: hemoglobin; Hct: hematocrit; MCV: mean corpuscular volume; MCH: mean corpuscular hemoglobin; MCHC: mean corpuscular hemoglobin concentration; RDW: red cell distribution width; Plt: platelet; HCO_3_: bicarbonate; CRP: C-reactive protein; AST: aspartate aminotransferase; ALT: alanine aminotransferase; GGT: gamma-glutamyl transferase; PT: prothrombin time; INR: international normalized ratio; PTT: partial thromboplastin time; *E. histolytica*: *Entamoeba histolytica*

Laboratory tests	Day one	Day two	Day three	Day four	Reference
WBC (10^9^/L)	8.39	5.07		4.75	5–15
Neutrophil (%)	84	72.6		47.7	
Lymphocyte (%)	7.21	13.23		30.75	
Monocyte (%)	8.32	12		18.11	
Eosinophil (%)	0.11	0.353		2.27	
Basophil (%)	0.455	1.83		01.149	
RBC (10^12^/L)	4.36	4.35		4.02	4.1–5.5
Hgb (g/dL)	12.4	12		11	11–14
Hct (%)	36.9	36		32.8	33–42
MCV (fL)	84.6	82.8		81.5	73–87
MCH (pg)	28.5	27.5		27.4	26–32
MCHC (g/dL)	33.6	33.3		33.6	30–37
RDW (%)	11.6	11.9		11.2	11.6–15.5
Plt (10^9^/L)	233	247		236	150–400
HCO_3_ (mEq/L)	17	20	19	26	20–28
CRP (mg/dL)	18	15.8	13.1		<5
Procalcitonin (ng/mL)			13.97	6.17	0–0.05
Urea (mmol/L)	3.6	2.2	2.0	<1.1	2.78–8.07
Creatinine (mg/dL)	40.6	32.4	26.6	23.5	28–52
AST (units/L)	2370	3394	969	307	5–34
ALT (units/L)	2038	3759	2834	1872	<55
Alkaline phosphatase (units/L)	162	151	145	145	142–335
GGT (IU/L)	36	70	60	59	9–36
Amylase (U/L)	127	133	264	198	25–125
Lipase (IU/L)	271	285	642	559	8–78
Albumin (g/L)	32	29	30	28	38–54
Total protein (g/L)	49	47	48	47	60–80
Total bilirubin (µmol/L)	14	9	12	10	3.4–20.5
Direct bilirubin (µmol/L)	8.3	7.8	6.3	5.6	<8.6
PT (s)		22.7	20.8	14.9	10.2–13.4
INR		2	1.82	1.28	0.9–1.1
PTT (s)		30.5	32.2	26.3	27–35
Urine ketone	+++	Negative	++		Negative
Urine nitrite	Negative	Negative	Negative		Negative
Urine leukocyte esterase	Negative		Negative		Negative
Stool parasitology	Negative	*E. histolytica*		Negative	
Urine culture	Negative	Negative	*Salmonella*		
Blood culture	Negative	Negative	Negative		
Stool culture			*Salmonella *species type D		

Blood culture returned negative on the second day of admission, and urine culture revealed *Salmonella *species, which was interpreted as sample contamination (Table 1). Therefore, urine analysis was repeated and was unremarkable. Stool culture was still pending, with another urine culture obtained for confirmation. Later during the day, the patient was in severe distress and developed excruciating epigastric and right upper quadrant abdominal pain. The possibility of perforated appendicitis complicated by abscess was considered and further investigations were ordered. The patient was started on cefotaxime, and a surgical consultation was obtained.

**Figure 1:**
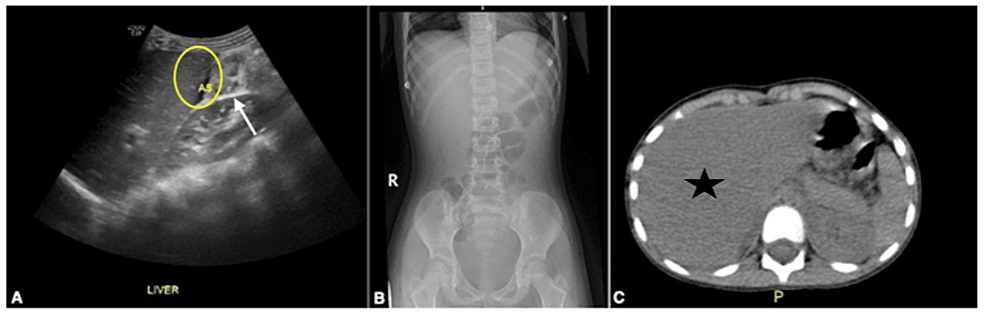
Abdominal US showing small ascites (circle) with edematous gallbladder wall thickening (arrow) (A). Abdominal X-ray showing no significant abnormality (B). CT abdomen with contrast depicting mild liver enlargement (star) and diffuse intestinal wall thickening (C). US: ultrasound; CT: computerized tomography

Complete blood count showed normal results. C-reactive protein (CRP) was significantly elevated with marked elevation of pancreatic and liver enzymes. Hepatitis serology was negative for hepatitis A, B, Epstein-Barr virus, and cytomegalovirus. Abdominal ultrasound showed ascites but was otherwise unremarkable (Figure 1A). Both chest and abdominal X-rays were unremarkable. Computerized tomography (CT) with contrast of the abdomen depicted mild liver enlargement and diffuse wall thickening in the small and large bowel loops (Figures 1B, 1C).

On the third day of admission, stool culture obtained on day two revealed *Salmonella *species type D. Laboratory investigations were repeated for comparison which indicated further derangement of pancreatic and liver enzymes. Inflammatory markers including procalcitonin and CRP were increased. Vitamin K was administered, and the patient was shifted to the pediatric intensive care unit (PICU). Following transfer to the PICU, the patient was kept on 10% dextrose until normalization of liver enzymes. A non-icteric picture of hepatitis was suspected, and the patient was kept on vitamin K 5 mg/kg/day.

**Figure 2:**
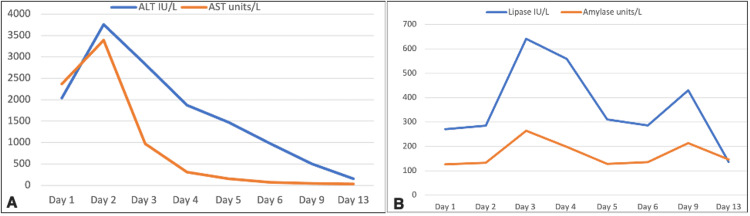
Trends of the liver (A) and pancreatic enzymes (B) over the full course of the patient’s hospitalization in days. AST: aspartate aminotransferase; ALT: alanine aminotransferase

On the fourth day, cefotaxime and metronidazole were continued. The patient was clinically improving and experienced no more symptoms. Daily laboratory investigations were obtained for comparison with similar trending. On the fifth day of admission, considering the improvement of the patient’s overall clinical picture and the near-normalization of the liver and pancreatic enzymes (Table 1, Figure 2), the PICU team decided to transfer her back to the ward and the plan was to continue ceftriaxone until day 10.

## Discussion

*Salmonella* infection is a major public health problem in the developing and underdeveloped world. Salmonellosis most frequently presents with acute enteritis, in which children develop signs of nausea, vomiting, bloody or mucogenic diarrhea, and abdominal discomfort in the right lower quadrant and periumbilical region. *Salmonella* gastroenteritis is often associated with fever and acute dehydration in older age groups of the pediatric population; however, the infection follows an afebrile course in neonates and younger infants [20].

The development of *Salmonella *gastroenteritis-related bacteremia is infrequent in children, but, if developed, Salmonella can spread and induce supportive focal lesions involving distant organs [20]. The disease is often associated with mild hepatomegaly and biochemical pancreatitis; however, severe hepatosplenomegaly and acute pancreatitis are aberrant findings in the pediatric population [21]. A study reported that the autopsy examination of patients who died from *Salmonella* food poisoning indicated profound disseminated interstitial pancreatitis [22], but pancreatitis might also be a result of multiorgan failure resulting from bacterial or endotoxin-induced shock. Several studies have investigated the development of *Salmonella* enteritis-related acute pancreatitis, but none of them provided clinically significant and clear evidence. A study reported that more than half of the Salmonella-infected patients developed adjuvant pancreatitis with mild-to-moderate symptoms [23], while another study showed mild elevation of serum amylase in one patient out of 51 *Salmonella*-infected patients and regarded it as a clinically insignificant finding [24].

Hermans et al. evaluated the pancreatic function of 14 *Salmonella*-infected hospitalized patients by assessing their serum amylase and lipase levels. In their study, seven patients had elevated enzymatic disturbance, and four patients had clinical signs of pancreatitis [25]. Baert et al. suggested the development of biochemical pancreatitis in 22% of *Salmonella *gastroenteritis patients [26]. The development of severe *Salmonella*-related hepatomegaly and acute pancreatitis is also related to the patient’s general physical health. A case report showed the development of severe *Salmonella *hepatitis in a young male with a history of occasional drinking and early-stage renal disease [27]. These results suggest that biological or clinical pancreatitis should be considered a crucial complication associated with *Salmonella *infection.

An experimental study analyzed the antibiogram of *S. enterica *serovar *typhi *and *S. enterica* serovar *paratyphi A*. The results indicated that both the serotypes were remarkably sensitive to chloramphenicol, cotrimoxazole, and ceftriaxone and eminently resistant to ciprofloxacin and nalidixic acid [28]. It was evident that chloramphenicol, cotrimoxazole, and ceftriaxone should be preferred in treating infections caused by *Salmonella* serotypes.

Our case describes a rare coexistence of *Salmonella* hepatitis and pancreatitis in a healthy child with no significant medical history. Different theories have been implicated in the pathogenesis of associated pancreatitis and hepatitis, of which hematogenous and lymphatic spread are currently the proposed mechanisms underlying pancreatitis. This case represented a convoluted approach to diagnosis as the patient was initially suspected of having foodborne illness evident by stool parasitology showing *Entamoeba histolytica* trophozoite, hence oral antiprotozoal was started. However, identifying *E. histolytica* trophozoites in the stool is highly unspecific to rule out a confirmed diagnosis. Both pathologic and non-pathologic strains of *E. histolytica* can colonize the intestinal surface and crypts of the colon, and the development of disease is associated with the erosion of intestinal mucosa. The severity of infection depends on the extent of erosion and the site of infection [29]. Nevertheless, the patient continued having severe epigastric and right upper quadrant abdominal pain aggravated by food intake, necessitating further investigations. As CT of the abdomen depicted hepatomegaly with rising pancreatic and liver function tests, it was imperative to think of non-icteric hepatitis causing liver failure due to fatty acid oxidation defect that was triggered by febrile illness. The differential diagnosis list narrowed after stool culture grew *Salmonella* type D and the patient’s improved clinical condition and normalization of laboratory markers after antibiotic treatment.

## Conclusions

In the pediatric population, the coexistence of acute pancreatitis and acute hepatitis as a complication of *Salmonella* infection have been scarcely reported in the literature. The presentation may be atypical, and a high level of clinical suspicion should arise once suspected. Early recognition and timely treatment halt the further progression and are associated with better outcomes.
